# The Impact of Stress Caused By Light Penetration and Agrotechnological Tools on Photosynthetic Behavior of Apple Trees

**DOI:** 10.1038/s41598-020-66179-3

**Published:** 2020-06-08

**Authors:** Kristina Laužikė, Vaida Sirgedaitė-Šėžienė, Nobertas Uselis, Giedrė Samuolienė

**Affiliations:** 1 Institute of Horticulture, Lithuanian Research Centre for Agriculture and Forestry, Kauno 30, Babtai Kaunas distr., Lithuania; 2 Institute of Forestry, Lithuanian Research Centre for Agriculture and Forestry, Liepų str. 1, LT-53101 Girionys Kaunas District, Lithuania

**Keywords:** Light responses, Photosystem II, Drought

## Abstract

The aim was to find out the impact of stress, caused by agrotechnological tools on photosynthetic behaviour of apple trees. The apple tree (*Malus domestica* Borkh.) cultivar Rubin was grafted on dwarfing rootstocks P60, planted in single rows spaced 1.25 m apart with 3.5 m between rows. In contrast to plant senescing reflectance index and nitrogen balance index, the photochemical reflectance index was significantly higher in 2018 compared with 2017. Such differences might be caused by drought stress on the summer and fast recovery before harvest time when measurements were made. The movement of nutrients and water disrupted by trunk incision had significantly negative effect on reflectance indices regardless on the year. Mechanical pruning with trunk incision and calcium-prohexadione lead to decreased dry to fresh weight ratio by 10–12% in first year of treatment. Mechanical pruning had significantly negative impact on photosynthetic rate, compared to pruning by super spindle it decreased 47%. Strong positive correlation between PRI and NBI 0,89–0,94, and strong negative correlations between PRI, NBI and PSRI −0.88 – (−0.91) were determined.

## Introduction

Not only environmental factors but also plant canopy ant light penetration into canopy strongly influence photosynthetic productivity and plants’ growth. Photosynthesis productivity depends on light, water, CO_2_, nutrients and even plant structure and architecture^1^ Light is important for all physiological processes in a plant. From 100% sun light only 40% is typical light interception by orchard systems and 15% (out of 40%) is limited by orchard design and leaf area^2^. During photosynthesis, solar energy binds to dry matter, so the photosynthetic behavior of the plant can be evaluated. Increasing photosynthesis goes to increase in biomass. Optimal plant photosynthesis system work is the main part of the biomass quantity^1,3^.

Non-destructive measurement methods easily measure variable optical properties without affecting the leaf at various stages of its development. In one measurement several functionally relevant leaf parameters is done without interruption^4,5^. Nitrogen (N) is one of the most important components of photosynthetic productivity is the also, it is a part of chlorophyll and it is one of the most important elements for biomass production^6^. The chlorophyll index (Chl) and the nitrogen balance index (NBI) measure the relative amount of chlorophyll and nitrogen at the same point on the leaf in the same moment and it may convey N dynamics in apple trees systems^7,8^. Plant synthesizes proteins and favors primary metabolism(nitrogen containing molecules) containing chlorophyll and just a few flavonols is shown by higher nitrogen balance index. NBI status of leaves has a relationship to light availability and light limitation as shown in literature^2,5,9^.

The Photochemical Reflectance Index (PRI), which is based on leaf reflectance at 531 nm, is suitable for tracking variations in photosynthetic activity from leaf^10^. PRI is commonly used as an appropriate indicator of abiotic factors that limit photosynthesis^11^. PRI via the xanthophyll cycle is related with photosystem II (PSII) and it is using as an alternative for light use efficiency. Photochemical reflectance index was applied as an active probe of pigment conversion, it can reflect almost all changes in the pigment content of the xanthophyll cycle^12^. Other authors have investigated that changes in pigment content and ratio due to leaf growth, aging, or chronic stress play an important role along with xanthophyll pigment epoxidation in the PRI signal. The photochemical reflectance index responds to the chlorophyll / carotenoid ratio in leaves due to species, age, and environmental conditions^5,12,13^. While PRI characterizes the photosynthetic efficiency, the plant senescing reflectance index (PSRI) is sensitive to the changes of carotenoids and chlorophyll ratio and it was used as a quantitative measure of leaf senescence. They are both commonly used to describe the changing physiological state of vegetation^13–15^. Reflectance spectra in the visible and near infra‐red range of the spectrum, acquired for maple (*Acer platanoides* L.), chestnut (*Aesculus hippocastanum* L.), potato (*Solanum tuberosum* L.), coleus (*Coleus blumei* Benth.), leaves and lemon (*Citrus limon* L.) and apple (*Malus domestica* Borkh.) were studied and it was found that leaf senescence effect is depending on pigment composition^14^.

Photosynthesis activity is associated not only with the yield of individual leaves, but also with the optimal use of light, considering the penetration of light through the canopy^16^. Alarcon and Sassenrath^17^ showed cotton leaf photosynthesis efficient dependence from shapes and canopy architecture. This shows that the effectiveness of photosynthesis can be controlled through the shape of the canopy and the best results were based on the high number of small-to-medium sized leaves. Like all plants, the ability of apple trees to sustain higher photosynthetic rates (P_N_) depends on the whole tree canopy and its regulation^16–18^. Also, Zhang *et al*.^19^ showed that photosynthesis consider from temperature, CO_2_ and especially light intensity and chlorophyll content. Moreover, photosynthetic rates are sensitive to various stresses like salt-alkaline, salt, water deficiency, shading and other^20^. One of the reasons for the P_N_ reduction is the stress of water drought and Ohashi *et al*. measured the decrease by up to 34%^21^. The inhibition of photosynthetic rate under water stress may be attributed not only to stomatal closure but also on several biochemical and photochemical processes^22,23^.

‘Rubin’ is large fruit variety. De Salvador *et al*.^24^ showed that heavy crop load decreased fruit size and weight. Denser canopy also has negative impact on fruit mass and yield^25^. Although calcium-prohexadione is using for smaller terminal growth it also has the impact for fruit weight. Usually fruit weight decreases by use of calcium-prohexadion. As Privé *et al*.^26^ showen Calcium-prohexadion can increase light penetration into the inner canopy of fully-grown apple trees and thus improve flower bud formation and expected yield.

Water and nutrients movement are disturbed by trunk girdling as well as by trunk incision. Trunk girdling and ringing significantly increase yield but most of the studies shown that photosynthetic behavior decreases in the same time as yields increase^27–29^. Trunk girdling not only increase fruit yield, but also can increase the influence of other agrotechnical tools using foliar spray or fertilization^29–32^.

Manual pruning of orchards requires a lot of resources and manpower to reduce the cost of gardening through mechanical pruning, trunk cutting, chemicals and more. Despite the amount of research on the relationship between the optical properties of apple leaves and the rate of photosynthesis in different apple agrotechnology tools, there is little. Thus, the aim was to elucidate the influence of stress caused by light penetration on canopy and agro-technological measures on photosynthetic behavior of apple trees.

## Materials and methods

### Plant material and growing conditions

A field experiment was carried out in an intensive orchard. The apple tree (*Malus domestica* Borkh.) cultivar Rubin was grafted on dwarfing rootstocks P60. Apple trees were planted in 2010 year in single rows spaced 1.25 m apart with 3.5 m between rows. Pest and disease management was carried out according to integrated plant protection practices and orchard was not irrigated. Soil conditions of the experimental orchard were following: clay loam, pH 7.3, humus 2.8%, P_2_O_5_ 255 mg kg^−1^, K_2_O 230 mg kg^−1^. Three single trees were fully randomized. The non-destructive and biometric analysis were performed in harvest time in the end of September. The samples were taken from five leaves in two levels: at 1.0–1.2 m and 1.8–2.0 m above the ground. Five agrotechnological tools were used: (1) each year hand pruning forming slender spindle (control); (2) mechanical pruning (each year) with hand pruning every second year; (3) mechanical pruning (each year); (4) trunk incision using chain saw +mechanical pruning (each year); (5) mechanical pruning (each year) +spraying with calcium-prohexadione. Mechanical pruning goes with hand pruning ones in three years removing the oldest branches from the stem base. Reducing the movement of water and nutrients by cutting the trunk also suppresses tree growth, reduces the number of shoots and at the same time increases the lighting of the canopy at the top of the it. Mechanical pruning, on the other hand, only shortens the branches, without the use of manual pruning, increasing the number of large branches, which increases the darkening of the crown. Also, trees grow actively in the upper part of the canopy, densify and multiply branches, which cast heavy shadows on the entire tree. Using only mechanical pruning, the trees resemble a hedge. While in the case of manual pruning, the large branches are removed from the trunk, there are no large branches at the top of the canopy, and the canopy is easily lightened and does not darken the rest of the crown.

### Photochemical reflectance index (PRI) and Plant senescing reflectance index (PSRI)

Spectral reflectance was measured using a leaf spectrometer (CID Bio-Science, USA) from 9 to 12 am. Reflection spectra obtained from the leaves were used to calculate photochemical reflectance index (PRI) and plant senescing reflectance index (PSRI). PRI shows changes in the xanthophyll cycle, using the following formula:

1 $${\rm{PRI}}=({{\rm{R}}}_{570}-{{\rm{R}}}_{531})/({{\rm{R}}}_{570}+{{\rm{R}}}_{531})$$

where R_570_ and R_531_ represent the leaf reflectance integrated over a 10 nm wavelength band centred on 570 and 531 nm, respectively

PSRI was proposed as being sensitive to the senescence phase of plant development and it’s calculated by formula:

2 $${\rm{PSRI}}=({{\rm{R}}}_{678}-{{\rm{R}}}_{500})/{{\rm{R}}}_{750}$$

where R_678,_ R_500_ and R_570_ represent the leaf reflectance integrated over a 10 nm wavelength band centred on 678, 500 and 570 nm, respectively

### Nitrogen balance index (NBI)

NBI was evaluated using no-destructive measurement of leaf chlorophyll and flavonoid content (related to Nitrogen/Carbon allocation) in the epidermis (Dualex ®4, USA).

Photosynthetic rate was determined at 9:00–12:00 am by using a LI-6400XT portable open flow gas exchange system (Li-COR Biosciences, Lincoln, USA). Reference air [CO_2_] (400 μmol mol^−1^), light intensity (1000 μmol m^−2^ s^−1^) and the flow rate of gas pump (500 mmol s ^−1^) were set.

### Biometric measurements

To determine the leaf area (cm^2^), twenty leaves were randomly sampled from all tree canopy and measured with a leaf area meter (AT Delta – T Device, UK). The dry mass of twenty leaves was determined by drying apple tree leaves in 70 °C for 48 h. (Venti cell 222, Medcenter Einrichtungen, Gräfeling, Germany) to constant weight^33^.

### Meteorogical conditions

**Figure 1:**
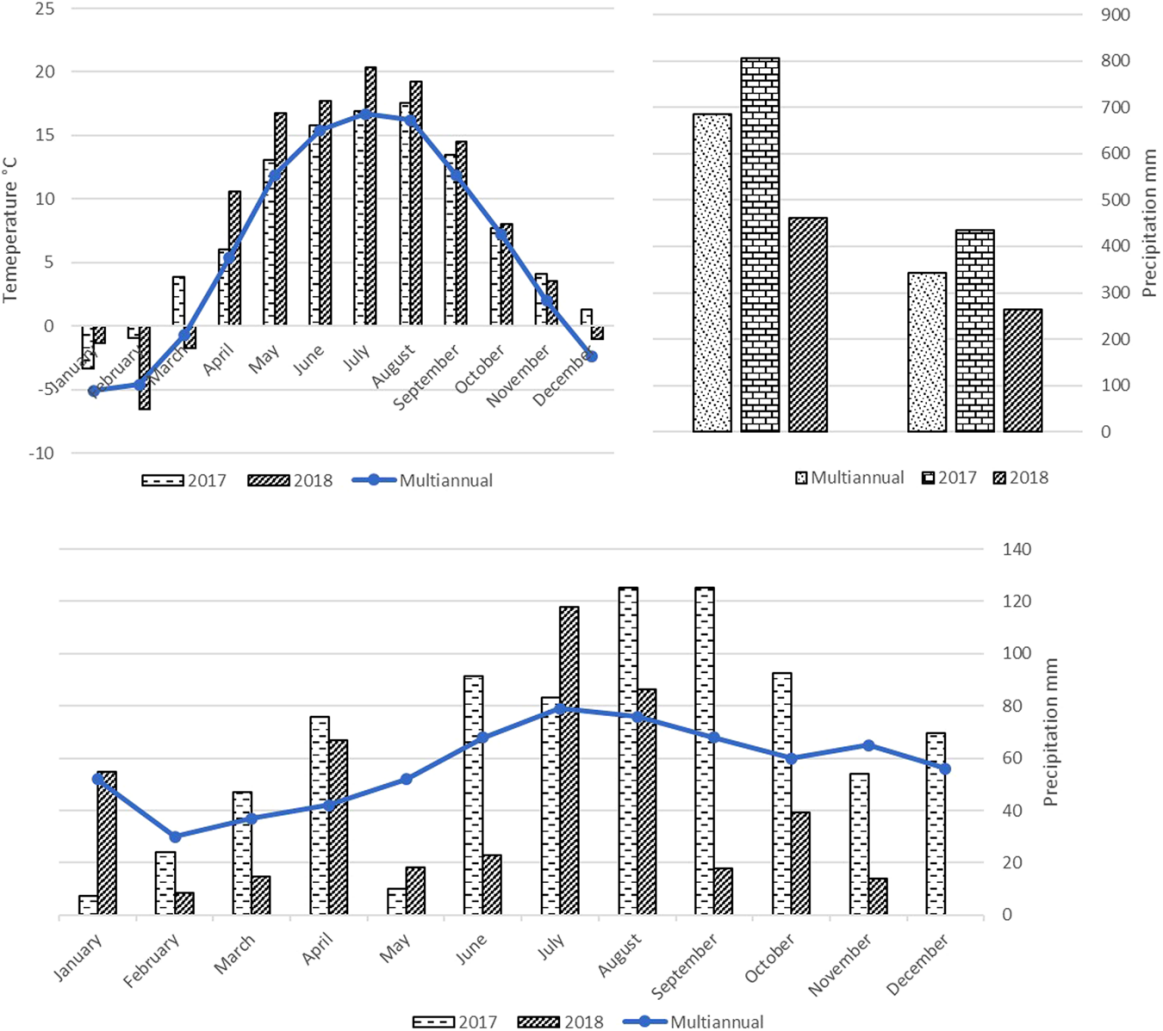
Meteorogical conditions in two years and the total precipitation on vegetation period and whole years compared to multiannual (average of 100 years) conditions.

The meteorogical data were collected from ‘iMetos’ meteorogical in an intensive orchard. The air temperature and precipitation in last two years were very variable (Fig. 1). Air temperature was close to multi-annual, while precipitation during the vegetation period (May to September) was much higher than multi-annual (average of 100 years), especially on harvest time in 2017. While during bloom period the precipitation was very low in both years. Drought year of research was characterized by a higher than usual temperature and low and very variable precipitation. Natural drought was announced in Lithuania that year (Lithuania, lat. 55°N, 2017–2018). Although rainfall in 2018 is significantly lower than in 2017 and average of multiannual precipitation, given that apple trees are perennial woody plants, we do not generally consider drought stress. However, on harvest time precipitation level was very low in 2018. On July 12, 2018, precipitation was 52 mm during one day, when on the other days it was only 0–8 mm per day.

### Statistical analysis

The data was processed using XLStat software (Addinsoft, 2019, USA, 2019), The data were processed using two-way ant three-way analysis of variance (ANOVA) Analysis of variance was performed with Duncan’s least significant difference test (P < 0.05).

## Results

**Figure 2:**
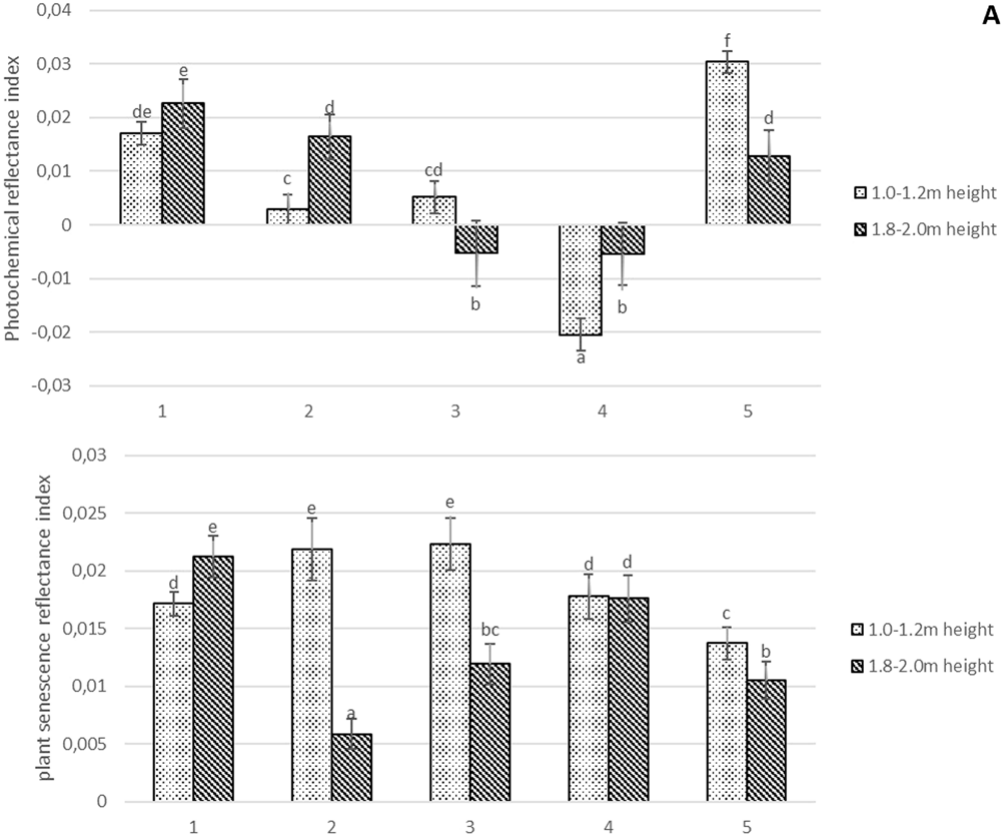
Photochemical reflectance (**A**) and plant senescence reflectance (**B**) indices in apple trees on harvest time in average of two years. 1. Each year hand pruning forming slender spindle (control); 2. Mechanical pruning (each year) with hand pruning every second year; 3. Mechanical pruning (each year); 4. Trunk incision using chain saw + mechanical pruning (each year); 5. Mechanical pruning (each year) + spraying with calcium-prohexadione. Averages followed by different letter within the same figure indicate significant differences according to the Duncan’s least significant difference test (P < 0.05). Error bars shows standard deviation.

Mechanical pruning results denser crown and light penetration into canopy. The lower light penetration into the crown resulted the decrease of PRI (25–100%) (Fig. 2A). However calcium-prohexadione increased light penetration into canopy at the same time increased PRI 45% compared to control. The movement of nutrients and water disrupted by trunk incision has significantly negative effect PRI. PRI was more than 50% lower by using trunk incision compared to control. Mechanical pruning increased PSRI to 30% in the lower part of the canopy, but decreased 50–65% in the top of the canopy (Fig. 2B).

**Figure 3:**
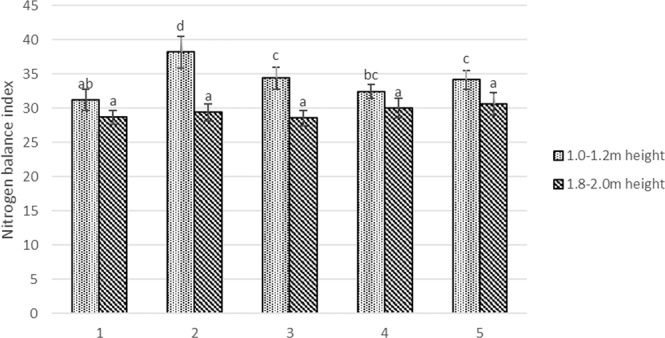
Nitrogen balance index in apple trees on harvest time. 1. Each year hand pruning forming slender spindle (control); 2. Mechanical pruning (each year) with hand pruning every second year; 3. Mechanical pruning (each year); 4. Trunk incision using chain saw + mechanical pruning (each year); 5. Mechanical pruning (each year) + spraying with calcium-prohexadione. Averages followed by different letter indicate significant differences according to the Duncan’s least significant difference test (P < 0.05). Error bars shows standard deviation.

Nitrogen balance index showed significant differences only in the lower part of canopy. Mechanical pruning with manual pruning increased NBI by 38% compared to control. Meanwhile, using trunk cut, the NBI variation is insignificant compared to the control (Fig. 3). NBI increased reached the highest values by the use of mechanical pruning with hand pruning in the lower part of the canopy in 2017 (Fig. S1)

**Figure 4:**
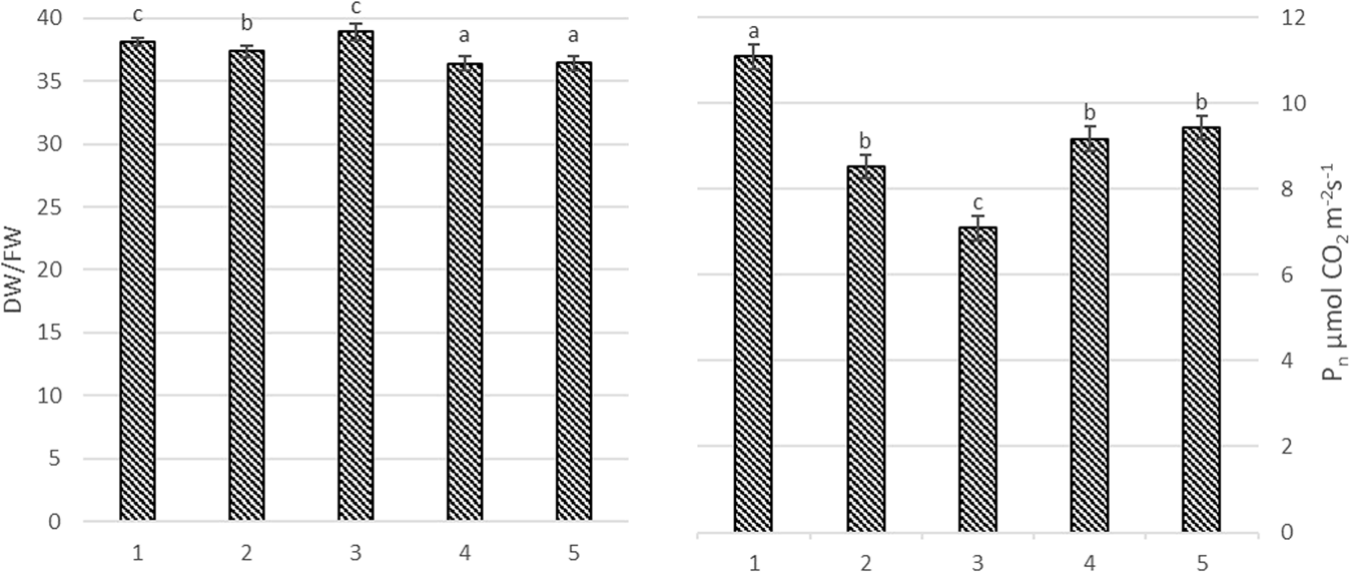
Dry and fresh weight ratio (**A**) and photosynthetic rate (**B**) in apple trees on harvest time. 1. Each year hand pruning forming slender spindle (control); 2. Mechanical pruning (each year) with hand pruning every second year; 3. Mechanical pruning (each year); 4. Trunk incision using chain saw + mechanical pruning (each year); 5. Mechanical pruning (each year) + spraying with calcium-prohexadione. Averages followed by different letter within the same figure indicate significant differences according to the Duncan’s least significant difference test (P < 0.05). Error bars shows standard deviation.

Trunk incision and calcium-prohexadione significantly decreased DW/FW ratio to 10–12% (Fig. 4). Compared to other agrotechnological tools, mechanical pruning resulted in significant decrease of photosynthetic rate (P_N_). P_N_ decreased by 47% using only mechanical pruning compared to pruning by super spindle. Mechanical pruning with hand pruning, trunk incision and calcium-prohexadione also resulted in significant decrease of P_N_ 10–20% compared to control.

**Figure 5:**
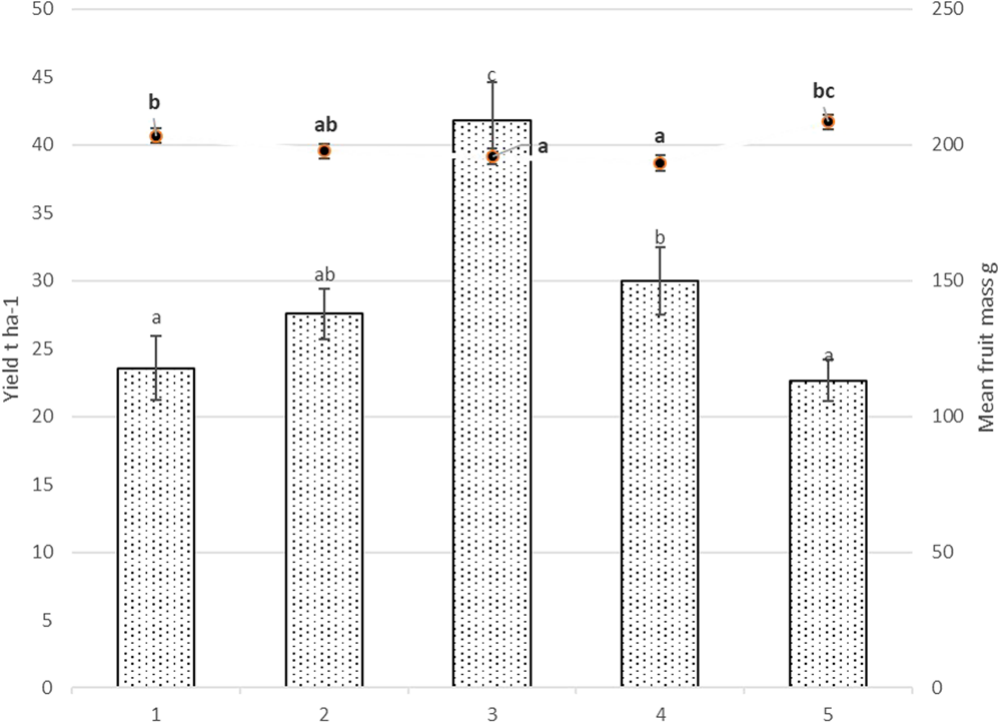
Total yield t/ha (columns) and mean apple weight g differences. 1. Each year hand pruning forming slender spindle (control); 2. Mechanical pruning (each year) with hand pruning every second year; 3. Mechanical pruning (each year); 4. Trunk incision using chain saw + mechanical pruning (each year); 5. Mechanical pruning (each year) + spraying with calcium-prohexadione Averages followed by different letter on yield and on mean fruit mass indicate significant differences according to the Duncan’s least significant difference test (P < 0.05). Error bars shows standard deviation.

Mechanical pruning increased apple yield by 45% but decreased fruit size by 4% in the average of two years (Fig. 5). Meanwhile trunk incision has no significant impact on yield but fruit mass decreased by 5.2% compared to control. Calcium-prohexadione has no significant effect for yield and fruit mass. There was no significant differences between mechanical pruning all treatment, however all treatments was significant higher compared to control in 2017. Meanwhile mechanical pruning reached 70 t ha^−1^ yield in 2018 (Fig. S2).

**Figure 6:**
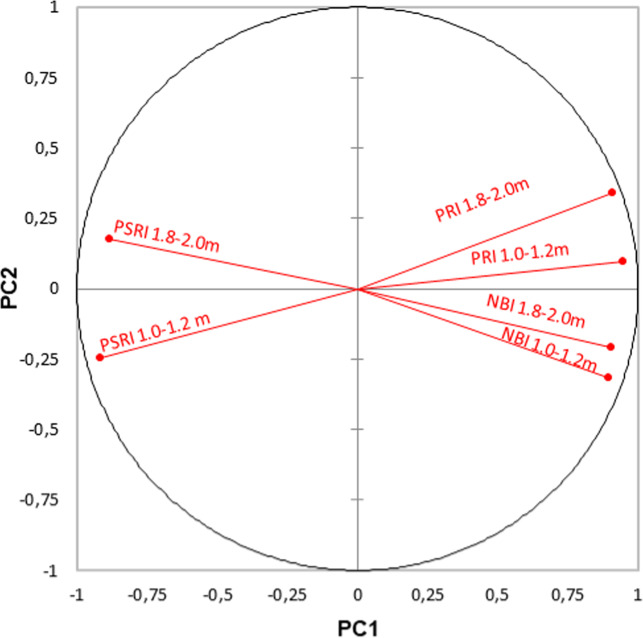
The principal components analysis (PCA) of the correlation of the indices of apple trees.

The treatment values were subjected to a principal component analysis (PCA). The first two components accounted for the majority of variation in the data set (88.59%). The variation of 82.60% could be explained by the first principal component (PC1) and 5.99% by the droughty principal component (PC2). Strong positive correlation between PRI and NBI 0,89–0.94, and strong negative correlations between PRI, NBI and PSRI −0.88 – (−0.91) were found (Fig. 6).

## Discussion

Wong *et al*.^34^ identified three causes of PRI variation, including changes in chlorophyll-carotenoid ratio (seasonality), light intensity, but also the effect of temperature on PRI (PRI and PSII efficiency). In their research is shown that at lower temperatures and dark green leaves, PRI values increase. Similarly, Weng *et al*.^35^ found that the relationship between the efficiency of PRS II and PRI varies with temperature and leaf color, they argue that lower PRI values are associated with increased illumination of mango tree leaves. While getting into the details of other authors’ data, and considering that this research comes from autumn measurements, when daytime temperatures drop, we find that during mechanical pruning, PRI responds more to temperature than to light intensity (Fig. 1). Mechanical pruning resulted in a denser apple tree shingle in the lower part of the tree, which caused the trap to release heat from the ground and prevent the wind from spreading. In addition, the leaves were light green due to poor lighting. Perhaps this is why PRI rates remained low at harvest (Fig. 2A). Optimal PSRI values ranged from −0.1 to 0.2^14^, and most trees were in good condition except for the trees treated by mechanical pruning with and without manual pruning. PSRI indices showed that the leaves of apple trees affected by mechanical pruning were did not reached the stage of aging at harvest time (Fig. 2B).

Denser planting of apple trees resulted less light penetration into canopy, thus lower NBI values were obtained^36^. Other studies we have done, and other authors have found that intense sunlight can reduce photosynthesis and promote metabolism^36,37^. Cronin and Lodge^37^ found that low light increased nitrogen content in leaf by 53%. In this study NBI decreased in top of the canopy by 25% (Fig. 3). Yang *et al*.^9^ demonstrated significant increases in N concentration in all *Abies fabri* (Mast.) organs by response to drought. We see that NBI increased significantly only in the sunny and droughty years (Fig. S1A,B)

Summer pruning and trunk incision is useful tool for better light penetration to overly dense canopies^2^. Meanwhile trunk incision disturbs water and nutrients movement and inhibits the vital functions and photosynthesis of the apple tree as decrease of photosynthetic rate was observed (Fig. 3).

Water deficit is one of the greatest limiting factor for photosynthesis and biomass accumulation^38–40^. The negative impact of the water deficit on woody plants has been extensively studied in trees (such as *Alnus glutinosa, Betula pendula, Cercis canadensis, Fraxinus excelsior Pinus sylvestris, Picea abies, Populus tremula, Quercus robur, Robinia pseudoacacia*)^41–43^. However, Kozlowski and Pallardy^44^ observations of woody plants adaptation to drought stress showed that trees have developed mechanisms to deal with dehydration conditions that are different from herbaceous plants. The amount of unstructured carbohydrates may also reflect tree drought, which may lead to a decrease in the concentration of unstructured carbohydrates, but as Gaul *et al*. In^45^, this usually only occurs in the later stages of drought.

Water intake increases with increased root fine growth, deep root formation, and solvent accumulation to reduce the water potential in the root tissue. Only then does the drought level become too severe, and the plants react by activating the mechanisms that protect tissues from cell damage, mainly by the action of protective solutes and proteins^46,47^. Trees that have been touched after a short drought have no major changes, even some tree species are able to recover quickly after a drought^48,49^. During treatment years was one drought episode form May till July (one day hard rainfall in July, and no rain other days), then another drought episode from the end of August till harvest time appeared. Apple trees were affected by short-term drought in 2018 but form the PRI and PSRI values it can be stated that the trees quickly recovered and functioned even better than in normal years.

Denser canopy formed by mechanical pruning decreases another limiting factor – light penetration into canopy. P_N_ is up to 50% lower in shade leaves compared to sun leaves^50^. Negative shade impact for trees photosynthesis also was described in several research like for *Acer rubrum* and *Betula papyrifera*^51^, *Fagus syltica*^52^, *Ginkgo biloba*^53^, *Malus domestica*^54^. However, shading can have positive impact for photosynthesis when it goes with drought but in our research there was no significant impact of shading^55^.

In agreement to Rze^56^ that calcium-prohexadione had no significant impact for fruit weight for apple cultivar ‘Rubin’ (Fig. 4). During the arid years calcium-prohexadione even increased ‘Rubin’ fruit weight. In our research the yields of the apple trees after prohexadione-Ca exposure were significantly bigger compared to untreated trees, meanwhile, other authors demonstrates similar results between treated and untreated trees^57,58^.

## Conclusion

Strong positive correlation between PRI and NBI 0,89–0,94, and strong negative correlations between PRI, NBI and PSRI −0.88 – (−0.91) were found. Trunk incision disturbed nutrients and water movement from roots to canopy that resulted the significant decrease of photochemical reflectance index, photosynthetic rate, fruit mass and accelerated leaf aging. The lower light penetration in denser canopy formed using mechanical pruning also resulted in significant decrease of photosynthetic indices (PRI, PSRI, P_n_). Mechanical pruning increased apple yield upto 45% but decreased fruit size by 4% in two years average. In general, the recommendation for industrial orchards is hand pruning every second year, mechanical pruning combined with reduced manual pruning lowered orchard maintenance but retained apple yield and photosynthetic activity.

## Supplementary information

Dataset 1.
