# Supplementary material for: The Impact of Stress Caused By Light Penetration and Agrotechnological Tools on Photosynthetic Behavior of Apple Trees
Source: Sci Rep. 2020 Jun 8;10:9177. doi: 10.1038/s41598-020-66179-3 (PMC7280272; doi:10.1038/s41598-020-66179-3)
Supplement: Supplementary file 1 — Dataset 1. [file 41598_2020_66179_MOESM1_ESM.docx]

**the impact of stress caused by LiGHT PENETRATION AND AGROTECHNOLOGICAL TOOLS on photosynthetic behavior of apple trees**

Kristina LAUŽIKĖ^*1^, Vaida SIRGEDAITĖ-ŠĖŽIENĖ^2^, Nobertas USELIS^1^, Giedrė SAMUOLIENĖ^1^

^1^Institute of Horticulture, Lithuanian Research Centre for Agriculture and Forestry, Kauno 30, Babtai, Kaunas distr., Lithuania

^2^Institute of Forestry, Lithuanian Research Centre for Agriculture and Forestry, Liepų str. 1, LT-53101 Girionys, Kaunas District, Lithuania

*Corresponding author: [K.Lauzike@lsdi.lt](mailto:K.Lauzike@lsdi.lt) ORCID ID 0000-0002-8821-1245

**Supplementary data.**


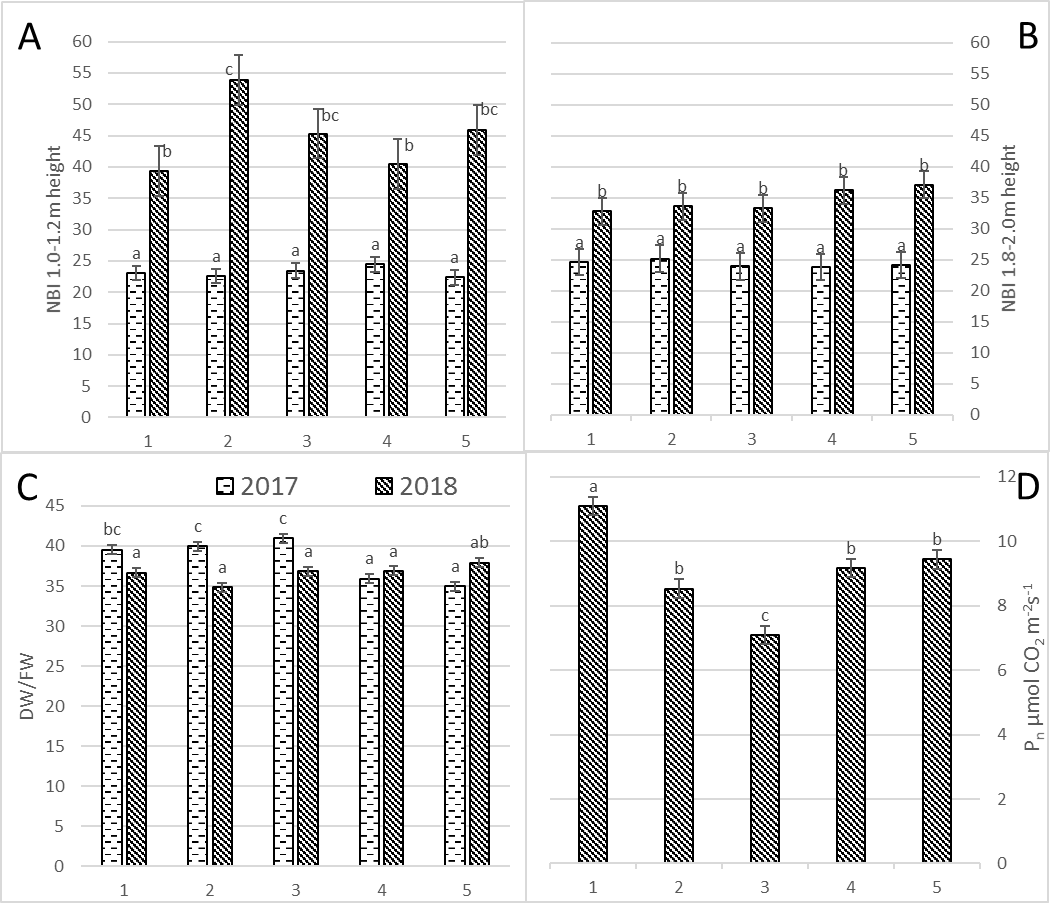


Fig. S1

Nitrogen balance index (NBI) (A – 1,8-2,0 m height, B – 1,0-1,2 m height), dry to fresh weight ratio (DW/FW) (C) and photosynthetic rate (P_N_) (D) in apple trees on harvest time during two years. 1. Each year hand pruning forming slender spindle (control); 2. Mechanical pruning (each year) with hand pruning every second year; 3. Mechanical pruning (each year); 4. Trunk incision using chain saw + mechanical pruning (each year); 5. Mechanical pruning (each year) + spraying with calcium-prohexadione. Averages followed by different letter within the same figure indicate significant differences according to the Duncan’s least significant difference test (P < 0.05).

Fig. S2

Variation of total yield t/ha (A) and apple weight g (B) during treatment years. 1. Each year hand pruning forming slender spindle (control); 2. Mechanical pruning (each year) with hand pruning every second year; 3. Mechanical pruning (each year); 4. Trunk incision using chain saw + mechanical pruning (each year); 5. Mechanical pruning (each year) + spraying with calcium-prohexadione Averages followed by different letter within the same figure indicate significant differences according to the Duncan’s least significant difference test (P < 0.05).
